# Effect of previous placenta previa on outcome of next pregnancy: a 10-year retrospective cohort study

**DOI:** 10.1186/s12884-020-02890-3

**Published:** 2020-04-15

**Authors:** Lizi Zhang, Shilei Bi, Lili Du, Jingjin Gong, Jingsi Chen, Wen Sun, Xinyang Shen, Jingman Tang, Luwen Ren, Guolu Chai, Zhijian Wang, Dunjin Chen

**Affiliations:** 1 Department of Obstetrics and Gynecology, Nanfang Hospital, Southern Medical University, 1838 Guangzhou Ave North, Guangzhou, 510515 China; 2 Department of Obstetrics and Gynecology, Guangzhou Medical Centre for Critical Pregnant Women, Key Laboratory for Major Obstetric Diseases of Guangdong Province, Third Affiliated Hospital of Guangzhou Medical University, 63 Duobao Road, Liwan District, Guangzhou, 510150 China

**Keywords:** Placenta previa, Cesarean delivery, Subsequent pregnancy, Pregnancy outcomes

## Abstract

**Background:**

To determine the effects of previous placenta previa on the maternal and neonatal outcomes of the next pregnancy.

**Methods:**

This 10-year retrospective cohort study was conducted in the Department of Obstetrics and Gynecology, Third Affiliated Hospital of Guangzhou Medical University, between January 2009 and 2018. We retrospectively analyzed the effects of a previous singleton pregnancy in women with and without placenta previa on the outcomes of the subsequent pregnancy. To control for confounders, we used multiple logistic regression models.

**Results:**

A total of 57,251 women with singleton pregnancies gave birth during the 10-year study period. Among them, 6070 women had two consecutive births. For the first pregnancy, 1603 women delivered by cesarean delivery and 4467 by vaginal delivery. Among women with a history of cesarean delivery, placenta previa was an independent risk factor for hemorrhage (adjusted odds ratio [aOR]: 2.25, 95% confidence interval [CI]: 1.1–4.62), placenta accreta spectrum (PAS) disorders (aOR: 4.11, 95% CI: 1.68–10.06), and placenta previa (aOR: 6.24, 95% CI: 2.85–13.67) during the subsequent pregnancy. Puerperal infection, blood transfusion, and perinatal outcomes did not significantly differ between women with a history of placenta previa and women without this history. Among women with a history of vaginal delivery, placenta previa increased the risk of PAS disorders (aOR: 5.71, 95% CI: 1.81–18.03) and placenta previa (aOR: 4.14, 95% CI: 1.07–16.04) during the subsequent pregnancy. There was no significant difference between the two groups in terms of hemorrhage, blood transfusion, puerperal infection, and perinatal outcomes.

**Conclusions:**

Women with a history of placenta previa are at risk for adverse outcomes such as postpartum hemorrhage, PAS disorders, and placenta previa in the subsequent pregnancy.

## Background

Placenta previa is a severe obstetric complication of pregnancy that occurs when the placenta attaches to the lower uterine segment and partially or completely covers the internal cervix [1, 2]. The prevalence of placenta previa is approximately 5 per 1000 pregnancies [3]. The incidence of placenta previa has increased in parallel with changing trends in risk factors [4], such as cesarean delivery, other uterine surgeries, advanced maternal age, high parity, smoking, cocaine use, and assisted reproductive technology (ART) [5–7].

Numerous studies have reported that placenta previa is associated with high maternal [8] and neonatal [9] adverse outcomes. Placenta previa is associated with an increased rate of cesarean delivery, hemorrhage, blood transfusion, as well as placenta accreta spectrum (PAS) disorders (which include placenta accreta, placenta increta, and placenta percreta), which can lead to hysterectomy, septicemia, intensive care unit (ICU) admission, thrombophlebitis, and even maternal death [7, 10–13]. The associated fetal complications mainly include fetal growth restriction and preterm delivery [14].

However, all of these complications caused by placenta previa lead to pathophysiological changes in the uterus, such as scar formation, endometrial damage, defective decidualization, and inflammation, which can potentially have an adverse effect on the outcome of the subsequent pregnancy. Furthermore, the risk factors for placenta previa may also influence the subsequent pregnancy. Some studies have reported that previous placenta previa is a risk factor for placenta previa in the subsequent pregnancy [15]. However, the impact of placenta previa on the outcomes of the subsequent pregnancy has not yet been fully explored.

With the adoption of the two-child family policy in China, some women with a history of placenta previa have given birth again. Therefore, this study aimed to determine whether a history of placenta previa was linked to adverse outcomes in the subsequent pregnancy.

## Methods

### Study design and subject selection

**Fig. 1:**
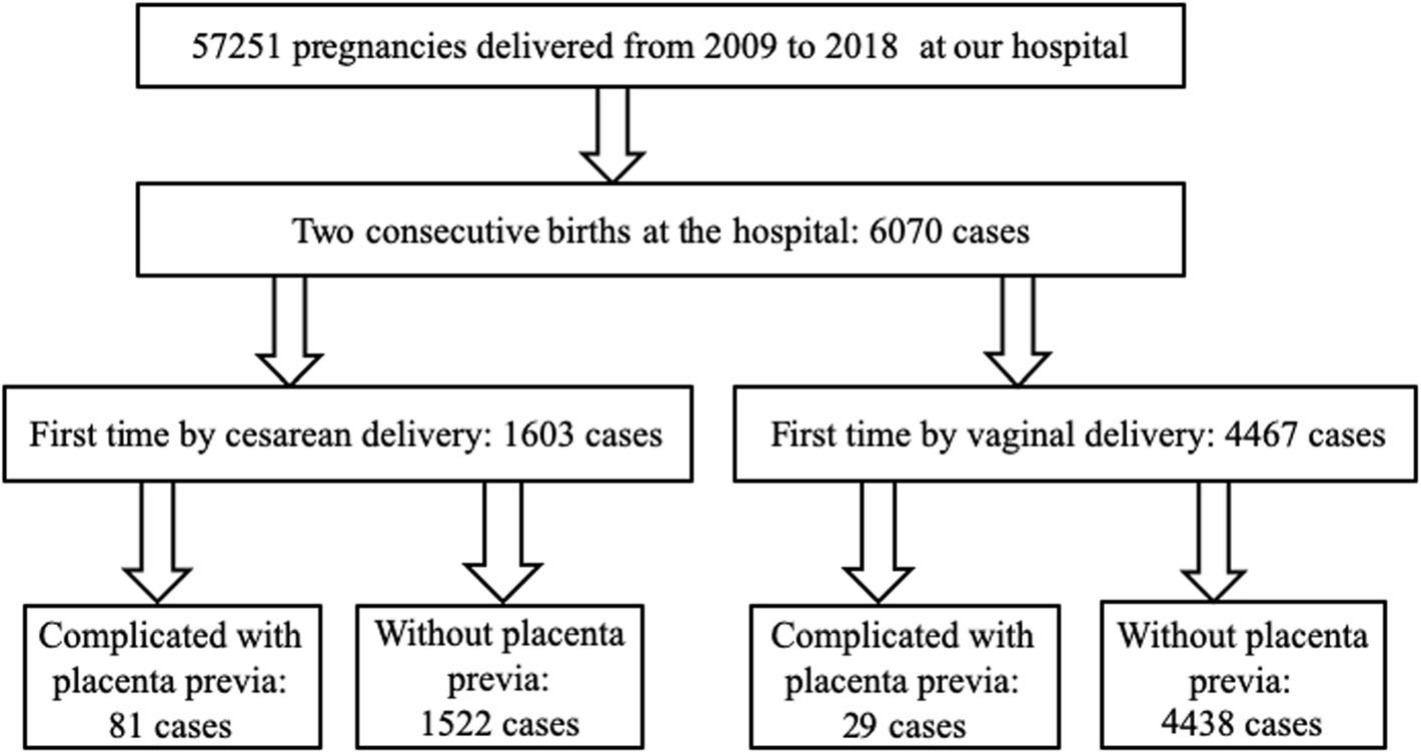
Flow diagram of the subject-enrollment process

This 10-year retrospective cohort study was conducted in the Department of Obstetrics and Gynecology, Third Affiliated Hospital of Guangzhou Medical University, Guangzhou Medical Centre for Critical Pregnant Women, Guangzhou, China, between January 2009 and January 2018. This study was approved by the Research Ethics Board of the Third Affiliated Hospital of Guangzhou Medical University. We reviewed the medical records of all women with two consecutive deliveries in our hospital. To account for the influence of the mode of the first delivery on the maternal and perinatal outcomes of the subsequent pregnancy, we divided the women into a cesarean delivery and a vaginal delivery group, according to the mode of delivery during the first pregnancy. Figure 1 shows a flow diagram of the subject-enrollment process. To identify the effects of placenta previa history on the outcomes of the subsequent pregnancy, we compared the women who had placenta previa during the first pregnancy with those who did not in both the cesarean delivery group and the vaginal delivery group.

Placenta previa was diagnosed using the last trans-abdominal or transvaginal ultrasonography performed before the delivery. All ultrasound examinations were performed by trained physicians. We used the criteria proposed by the multidisciplinary workshop of the American Institute of Ultrasound in Medicine for the diagnosis of placenta previa [16]. According to these criteria, the term placenta previa is used when the placenta lies directly over the internal os. For pregnancies greater than 16 weeks of gestation, the placenta is called as ‘low lying’ when the placental edge is less than 20 mm from the internal os. Women in whom the placenta moved away from the cervix with the progression of the pregnancy were excluded.

### Maternal clinical characteristics

The medical records of each subject were reviewed, and the following details were recorded: gestational age, maternal age, mode of conception (natural vs. assisted), mode of delivery (vaginal vs. cesarean delivery), number of abortions and vaginal deliveries, level of education, time interval between two deliveries, and sex of the offspring.

### Outcome measures

We created a composite outcome variable to evaluate adverse maternal and perinatal outcomes separately. Adverse maternal outcomes included any of the following: postpartum hemorrhage (PPH), blood transfusion, PAS disorders (placenta accreta, placenta increta, and placenta percreta), placenta previa, hysterectomy, ICU admission, puerperal infection, and maternal mortality. We defined postpartum hemorrhage as the loss of > 500 mL blood after a vaginal delivery or > 1000 mL blood after a cesarean delivery [17]. PAS disorders were diagnosed using the intraoperative findings or postoperative pathology. Adverse neonatal outcomes included 1-min 5-min and 10-min Apgar scores ≤7, prematurity (< 37 weeks), low birth weight (< 2500 g), perinatal mortality and stillbirth.

### Statistical analysis

Statistical analysis was performed using SPSS *v*21.0 for Mac. The chi-square test was used to compare categorical variables. The nonparametric Mann–Whitney *U*-test was used to compare continuous variables. Potential confounders considered were maternal age, gestational weeks, level of education, mode of delivery and conception, number of abortions and prior vaginal deliveries, and the time interval between the two deliveries. A multivariate analysis was performed to determine the role of a history of placenta previa in adverse maternal and perinatal outcomes of the subsequent pregnancy. Crude odds ratios (ORs) and adjusted odds ratios (aORs), along with their 95% confidence intervals (CIs), were calculated. Differences with *P*-values of < 0.05 were considered to be statistically significant.

## Results

### Maternal clinical characteristics

A total of 57,251 women with singleton pregnancies gave birth in the Department of Obstetrics and Gynecology, Third Affiliated Hospital of Guangzhou Medical University, between 2009 and 2018. Among them, 6070 women had two consecutive births in our hospital. During the first pregnancy, 1603 women delivered by cesarean delivery, and 4467 women delivered by vaginal delivery. The first pregnancy was complicated by placenta previa in 81 women in the cesarean delivery group and 29 women in the vaginal delivery group (Fig. 1). Tables 1 and 2 show the clinical characteristics of the patients in the cesarean delivery and vaginal delivery groups, respectively. In the cesarean delivery group, women with a history of placenta previa were significantly older (32.17 ± 3.99 vs. 33.64 ± 3.83 years, *P* < 0.05) and had a significantly lower educational level (*P* < 0.05) than the women without a history of placenta previa. The mode of conception, gestational weeks, number of abortions and prior vaginal deliveries, time interval between the two deliveries, and sex of the newborn were similar in women with and without placenta previa (Table 1). In the vaginal delivery group, the rate of ART and the number of vaginal deliveries were significantly higher (10.3% vs. 2.3, 24.1% vs. 10%, *P* < 0.05) and the number of gestational weeks (37.45 ± 4.18 vs. 38.57 ± 2.3 weeks) and time interval between the two deliveries were significantly lower (2.62 ± 1.47 vs. 3.23 ± 1.76 years, *P* < 0.05) among women with a history of placenta previa than among women without this history. Maternal age, level of education, number of prior abortions, and sex of the newborn were similar in women with a history of placenta previa and women without this history (Table 2).

**Table 1:** Clinical characteristics of women in the cesarean delivery group

Characteristics	Without PP history (***n*** = 1522)	With PP history (***n*** = 81)	***P*** value
Maternal age (years)	32.17 ± 3.99	33.64 ± 3.83	< 0.05^a^
< 35 years	1095 (71.9%)	48 (59.3%)	
≥ 35 years	427 (28.1%)	33 (40.7%)	
Gestational age	38.19 ± 2.22	37.94 ± 2.45	0.553^a^
< 28 weeks	18 (1.2%)	2 (2.5%)	
28–34 weeks	31 (2%)	1 (1.2%)	
34–37 weeks	78 (5.1%)	6 (7.4%)	
≥ 37 weeks	1394 (91.7%)	72 (88.9%)	
Level of education			< 0.05
College	957 (62.9%)	39 (48.1%)	
Below college	534 (35.1%)	37 (45.6%)	
Unknown	31 (2%)	5 (6.2%)	
Mode of conception			0.837
Natural	1478 (97.1%)	78 (96.3%)	
ART	44 (2.9%)	3 (3.7%)	
Prior abortions			0.533
0	1012 (66.5%)	52 (64.2%)	
1	344 (22.6%)	23 (28.4%)	
≥ 2	161 (10.6%)	6 (7.4%)	
Prior deliveries			0.632
0	1442 (94.7%)	75 (92.6%)	
1	71 (4.7%)	5 (6.2%)	
≥ 2	9 (0.6%)	1 (1.2%)	
Sex of offspring			0.824
Male	796 (52.3%)	45 (55.6%)	
Female	726 (47.7%)	36 (44.4%)	
Interval time	4.08 ± 1.75	3.96 ± 2.01	0.564

*PP* placenta previa; *ART* assisted reproductive technology

^a^: The Mann–Whitney *U*-test was used to calculate *P* values for continuous variables

**Table 2:** Clinical characteristics of women in the vaginal delivery group

Characteristics	Without PP history (***n*** = 4438)	With PP history (***n*** = 29)	***P*** value
Maternal age (years)	30.39 ± 4.07	32.41 ± 4.32	0.42^a^
< 35 years	3760 (84.7%)	23 (79.3%)	
≥ 35 years	678 (15.3%)	6 (20.7%)	
Gestational age	38.57 ± 2.3	37.45 ± 4.18	< 0.05^a^
< 28 weeks	55 (1.2%)	1 (3.4%)	
28–34 weeks	56 (1.3%)	3 (10.3%)	
34–37 weeks	194 (4.4%)	1 (3.4%)	
≥ 37 weeks	4130 (93.1%)	24 (82.8%)	
Level of education			0.067
College	2701 (60.9%)	13 (44.8%)	
Below college	1629 (36.7%)	14 (48.3%)	
Unknown	108 (2.4%)	2 (6.9%)	
Mode of conception			< 0.05
Natural	4337 (97.7%)	26 (89.7%)	
ART	101 (2.3%)	3 (10.3%)	
Prior abortions			0.991
0	3222 (72.6%)	21 (72.4%)	
1	886 (20%)	6 (20.7%)	
≥ 2	330 (7.4%)	2 (6.9%)	
Prior deliveries			< 0.05
1	3992 (90%)	22 (75.9%)	
≥ 2	346 (10%)	7 (24.1%)	
Sex of offspring			0.783
Male	2334 (52.5%)	18 (62.1%)	
Female	2104 (47.4%)	11 (37.9%)	
Interval time	3.23 ± 1.76	2.62 ± 1.47	< 0.05

*PP* placenta previa; *ART* assisted reproductive technology

^a^: Mann–Whitney *U*-test was used to calculate *P* values for continuous variables

### Multivariate analysis

Tables 3 and 4 show the results of the multivariate analysis for the composite pregnancy outcomes. A history of placenta previa was found to be an independent risk factor for PAS disorders (cesarean delivery group: aOR, 4.11; 95% CI, 1.68–10.06; vaginal delivery group: aOR, 5.71; 95% CI, 1.81–18.03) and placenta previa (cesarean delivery group: aOR, 6.24; 95% CI, 2.85–13.67; vaginal delivery group: aOR, 4.14; 95% CI, 1.07–16.04) in the subsequent pregnancy, regardless of the mode of previous delivery. In the cesarean delivery group, a history of placenta previa increased the risk of PPH (aOR: 2.25, 95% CI: 1.1–4.62), but did not influence the rate of puerperal infection or the perinatal outcomes (Table 5). The rate of blood transfusion was higher in women with a history of placenta previa than in women without this history (cesarean delivery group: 6.2% vs. 2.1%, vaginal delivery group: 6.9% vs. 1.1%, *P* < 0.05). However, after adjustments for cofounders, placenta previa history did not increase the risk of blood transfusion in the two groups. In the vaginal delivery group, placenta previa history did not affect the perinatal outcomes after adjustments for cofounders, although the rate of cesarean delivery during the second pregnancy (31% vs. 8.6%), the number of newborns with a birth weight < 2500 g (24.1% vs. 6.6%), and the number of preterm deliveries (17.2% vs. 6.9%) were higher among women with a history of placenta previa than among women without this history (Table 6).

**Table 3:** Pregnancy outcomes of women in the cesarean delivery group

Variable	Without PP history (***n*** = 1522)	With PP history (***n*** = 81)	OR	95% CI	aOR	95% CI
Cesarean delivery	1259 (82.7%)	68 (84%)	1.09	0.6–2.01	1.13	0.59–2.13
PPH	89 (5.8%)	10 (12.3%)	2.27	1.13–4.55	2.25	1.1–4.62
PAS	30 (2%)	7 (8.6%)	4.71	2–11.06	4.11	1.68–10.06
Transfusion	32 (2.1%)	5 (6.2%)	3.06	1.16–8.08	2.73	0.98–7.53
ICU	8 (0.5%)	0				
Hysterectomy	1 (0.1%)	0				
Maternal mortality	0	0				
PP	23 (2.1%)	10 (12.3%)	6.56	3.1–13.87	6.24	2.85–13.67
Puerperal infection	62 (4.1%)	2 (2.5%)	0.6	0.14–2.48	0.61	0.15–2.58

*OR* odds ratio; *aOR* adjusted odds ratio; *CI* confidence interval; *ICU* intensive care unit; *PP* placenta previa; *PPH* postpartum hemorrhage; *PAS* placenta accreta spectrum disorders

**Table 4:** Pregnancy outcomes of women in the vaginal delivery group

Variable	Without PP history (***n*** = 4438)	With PPhistory (***n*** = 29)	OR	95% CI	aOR	95% CI
Cesarean delivery	383 (8.6%)	9 (31%)	4.76	2.15–10.54	2.27	0.88–5.82
PPH	319 (7.2%)	3 (10.3%)	1.49	0.45–4.95	1.27	0.37–4.35
PAS	83 (1.9%)	4 (13.8%)	8.4	2.8–264.66	5.71	1.81–18.03
Transfusion	47 (1.1%)	2 (6.9%)	6.92	1.6–29.94	3.3	0.66–16.6
ICU	2 (0)	0				
Hysterectomy	0	0				
Maternal mortality	0	0				
PP	61 (1.4%)	3 (10.3%)	8.28	2.44–28.09	4.14	1.07–16.04
Puerperal infection	32 (0.7%)	0				

*OR* odds ratio; *aOR* adjusted odds ratio; *CI* confidence interval; *ICU* intensive care unit; *PP* placenta previa; *PPH* postpartum hemorrhage; *PAS* placenta accreta spectrum disorders

**Table 5:** Perinatal outcomes in the cesarean delivery group

Outcome	Without PP history	With PP history	OR	95% CI	aOR	95% CI
Stillbirth	28 (1.8%)	2 (2.5%)	1.35	0.32–5.77	1.39	0.16–12.44
Perinatal mortality	3 (0.2%)	0				
Apgar 1 min ≤ 7	53 (3.5%)	3 (3.7%)	1.07	0.33–3.49	0.71	0.13–3.91
Apgar 5 min ≤ 7	38 (2.5%)	2 (2.5%)	1.08	0.25–4.55	0.52	0.06–4.43
Apgar 10 min ≤ 7	35 (2.3%)	2 (2.5%)	0.72	0.09–5.88	0.78	0.1–6.09
Preterm delivery (< 37 weeks)	127 (8.3%)	9 (11.1%)	1.37	0.67–2.8	1.25	0.6–2.59
Low birth weight (< 2500 g)	124 (8.2%)	5 (6.2%)	0.74	0.29–1.87	0.34	0.08–1.47

*PP* placenta previa; *OR* odds ratio; *aOR* adjusted odds ratio; *CI* confidence interval

**Table 6:** Perinatal outcomes in the vaginal delivery group

Outcome	Without PP history	With PP history	OR	95% CI	aOR	95% CI
Stillbirth	89 (2%)	2 (6.9%)	3.62	0.85–15.46	0.34	0.04–2.76
Perinatal mortality	2 (0)	0				
Apgar 1 min ≤ 7	58 (1.3%)	1 (3.7%)	2.75	0.37–20.6	1.32	0.15–11.89
Apgar 5 min ≤ 7	9 (0.2%)	0				
Apgar 10 min ≤ 7	5 (0.1%)	0				
Preterm delivery (< 37 weeks)	305 (6.9%)	5 (17.2%)	2.82	1.07–7.45	1.51	0.53–4.27
Low birth weight (< 2500 g)	293 (6.6%)	7 (24.1%)	4.76	2.15–10.54	2.27	0.88–5.82

*PP* placenta previa; *OR* odds ratio; *aOR* adjusted odds ratio; *CI* confidence interval

## Discussion

A previous study has reported that the mode of delivery significantly influences the perinatal outcomes of the subsequent pregnancy [18]. To better understand the effect of previous placenta previa on the outcome of the next pregnancy, we divided the women into a cesarean delivery group and a vaginal delivery group, according to the mode of delivery during the first pregnancy. The present study showed that a history of placenta previa was an independent risk factor for PAS (aOR: 4.11, 95% CI: 1.68–10.06; aOR: 5.71, 95% CI: 1.81–18.03) and placenta previa (aOR: 6.24, 95% CI: 2.85–13.67; aOR: 4.14, 95% CI: 1.07–16.04) during the subsequent pregnancy in both the cesarean and vaginal delivery groups, respectively. Furthermore, in the cesarean delivery group, placenta previa history increased the risk of postpartum hemorrhage (aOR: 2.25, 95% CI: 1.1–4.62) during the subsequent pregnancy.

The incidence rate of placenta previa in our study, i.e., 4.9% (2831/57,251), is considerably high. Rosenberg et al. conducted a population-based study consisting of 185,475 singleton pregnancies, and found that the incidence rate of placenta previa was 0.49% [12]. In a cross-sectional study, placenta previa complicated 625 of 249,476 (2.50/1000) singleton births among nulliparous women and 915 of 347,086 (2.64/1000) singleton births among multiparous women [19]. As our center is a tertiary hospital specializing in the treatment of women with pregnancy-related illnesses, many of our patients were pregnant women with severe complications who were referred from other areas. The data of this unique patient cohort allowed us to investigate the impact of prior placenta previa on the outcomes of the subsequent pregnancy. Our data are comparable to those of West China Second University Hospital, Sichuan University, which reported an incidence rate of placenta previa of 4.84% (3840/79,304, 11]. The incidence of placenta previa depends on the diagnostic criteria used, the use of transvaginal ultrasonography, and the rising risk factors. Thus, the number of cases of placenta previa and its complications will continue to increase.

With the adoption of the two-child family policy in China, the number of multiparous women has been increasing in recent years. We found that a history of placenta previa was an independent risk factor for adverse outcomes in the subsequent pregnancy. The risk of PAS disorders and placenta previa were higher in women with a history of placenta previa than in women without this history, regardless of the mode of delivery. Anderson-Bagga et al. reported that prior placenta previa was a risk factor for placenta previa during subsequent pregnancies [15]. Placenta accreta and placenta previa have some common risk factors, such as disruption of the uterine endometrium, manual removal of the placenta, and uterine scarring due to intrauterine or intraoperative procedures or other gynecological surgeries [20]. Jauniaux et al. reported that the complication of placenta previa was present in more than 90% of patients with placenta accreta [12, 21]. A placenta located in the lower segment of the uterus influences the contraction of the uterus and its ability to compress the blood vessels, leading to bleeding and more intrauterine intraoperative procedures [22], which in turn result in uterine scar formation and pelvic adhesions. The area of scarring will exhibit poor vascularization, deficient tissue oxygenation, and inflammation, which may inhibit re-epithelialization and decidualization, leading to abnormal placental anchoring villi and invasion of trophoblasts [23]. Inappropriate implantation and placental development have been found during the subsequent pregnancy in women with a history of placenta previa [20].

In our study, the risk of PPH in women with a history of placenta previa was 2.25 times that in women without this history in the cesarean delivery group, but in the vaginal delivery group, a history of placenta previa did not increase the risk of PPH. In the cesarean delivery group, in most patients with placenta previa, the placenta covered the internal os partially or completely; moreover, the placenta in the lower uterine segment became larger and invaded deeper to ensure blood supply [8]. During the cesarean delivery, more intrauterine procedures were needed in these patients, and the damage to the uterus was severe. The muscular layer around the scar area was lacking or intermittent, which impaired the contraction of the uterus and the compression of the blood vessels. Moreover, the rates of placenta accreta and placenta previa in the subsequent pregnancy increased, as mentioned earlier. Furthermore, an abnormally invasive placenta can result in life-threatening hemorrhage as the placental separation proceeds [24]. A history of placenta previa did not increase the risk of blood transfusion, because the effect of previous placenta previa on PPH may be moderate. Many women complicated with PPH did not need blood transfusion.

Puerperal infection is a complication in women with placenta previa and repeated antepartum hemorrhage [25], and can lead to endometritis. The metabolic environment and inflammation affect endometrial receptivity, leading to inadequate uterine contractility and progesterone resistance [26]. The inflammatory mediators and oxidative stress will result in defective decidualization and remodeling of the uterine spiral vessels. Furthermore, in the lower segment of the uterus, anomalous blastocyst implantation and dysperistalsis of uterine contraction caused by pelvic adhesions [27] may result in placenta previa. However, in this study, a history of placenta previa did not increase the rate of puerperal infection in the subsequent pregnancy. The underlying mechanisms are still unknown. The relationship between endometritis and placenta previa history needs to be studied further.

In this study, the incidences of placenta previa and PAS in the subsequent pregnancy were higher in women with a history of placenta previa than in women without this history. Some studies have reported that abnormal placentation and poor blood supply can decrease the placental weight, and influence fetal oxygenation and growth [8]. However, in this study, there was no significant difference in the outcomes of the newborns among women with and without placenta previa. The association between placenta previa and fetal growth restriction is controversial [9, 28]. A meta-analysis showed that placenta previa was associated with a mild increase in the risk of intrauterine growth restriction [29]. Jauniaux et al. found no difference in fetal growth between women with placenta previa and PAS disorders and women with placenta previa only [30]. The effects of placenta previa on the neonatal outcomes in subsequent pregnancies have seldom been reported. More studies are therefore needed.

Our study has certain limitations. The present study was a single-center, retrospective study. Our center is a tertiary hospital specializing in the treatment of women with pregnancy-related illnesses who were referred from other areas. Thus, this study was vulnerable to referral bias. On the basis of the present results, it is difficult to prove the mechanism explaining why pregnant women with a history of placenta previa were at risk for adverse outcomes in the subsequent pregnancy. Future studies should examine the effects of a history of placenta previa on the outcomes of subsequent pregnancies in other population-based samples.

## Conclusions

In conclusion, the current study of a cohort of singleton births from our center showed that pregnant women with a history of placenta previa were at risk for adverse outcomes such as postpartum hemorrhage, PAS disorders, and placenta previa in the subsequent pregnancy. We recommend that obstetricians advise such patients about the risks involved and undertake careful perinatal surveillance [31] for these patients.

## Data Availability

The datasets used and/or analyzed during the current study are available from the corresponding author on reasonable request.

## Abbreviations

aOR: Adjusted odds ratio
CI: Confidence interval
PP: Placenta previa
ART: Assisted reproductive technology
PPH: Postpartum hemorrhage
PAS disorders: Placenta accreta spectrum disorders (placenta accreta, placenta increta, and placenta percreta)
ICU: Intensive care unit
